# Active In-Database Processing to Support Ambient Assisted Living Systems

**DOI:** 10.3390/s140814765

**Published:** 2014-08-12

**Authors:** Wagner O. de Morais, Jens Lundström, Nicholas Wickström

**Affiliations:** School of Information Science, Computer and Electrical Engineering, Halmstad University, Box 823, Halmstad 30118, Sweden; E-Mails: jens.lundstrom@hh.se (J.L.); nicholas.wickstrom@hh.se (N.W.)

**Keywords:** healthcare technology, smart homes, ambient assisted living, database management systems, active databases, in-database processing, data mining

## Abstract

As an alternative to the existing software architectures that underpin the development of smart homes and ambient assisted living (AAL) systems, this work presents a database-centric architecture that takes advantage of active databases and in-database processing. Current platforms supporting AAL systems use database management systems (DBMSs) exclusively for data storage. Active databases employ database triggers to detect and react to events taking place inside or outside of the database. DBMSs can be extended with stored procedures and functions that enable in-database processing. This means that the data processing is integrated and performed within the DBMS. The feasibility and flexibility of the proposed approach were demonstrated with the implementation of three distinct AAL services. The active database was used to detect bed-exits and to discover common room transitions and deviations during the night. In-database machine learning methods were used to model early night behaviors. Consequently, active in-database processing avoids transferring sensitive data outside the database, and this improves performance, security and privacy. Furthermore, centralizing the computation into the DBMS facilitates code reuse, adaptation and maintenance. These are important system properties that take into account the evolving heterogeneity of users, their needs and the devices that are characteristic of smart homes and AAL systems. Therefore, DBMSs can provide capabilities to address requirements for scalability, security, privacy, dependability and personalization in applications of smart environments in healthcare.

## 1. Introduction

Storage is an important and required functionality in continuous, long-term, home-based monitoring systems, and the database management system (DBMS) is the most common, but not fully exploited, component among software architectures underpinning smart environments, such as smart homes, and ambient assisted living (AAL) systems.

As an extension [1] and an alternative to existing platforms supporting the development of smart homes and AAL systems, this work proposes a database-centric architecture that explores the capabilities of DBMSs beyond those of data management.

### 1.1. Background

Home care has been suggested to be a sustainable alternative to traditional care, because it has the potential to prevent unnecessary acute or long-term institutionalization and to enable individuals to stay in their homes and communities for as long as possible [2]. Similar to other countries in Europe, in Sweden, county councils and municipalities provide medical, social and personal care services for care beneficiaries in their own homes. Most people receiving home care services are old or disabled individuals living alone.

Home care visits are normally planned, but when social alarm devices are offered to care beneficiaries, unplanned emergency response visits also occur. A social alarm is a portable device and includes a push-button to alert a care unit. Social alarms can include also a movement sensor that automatically triggers an alarm upon inactivity. The device is commonly worn as a wrist-watch or as a pendant necklace.

Although most home care services are provided during the daytime, some individuals require assistance during the night. In a nighttime visit, a night patrol team can help with medication, diapering, toileting and repositioning in bed [3]. However, supervision visits are more common, *i.e.*, the night patrol team, without waking up the resident, checks if the person is in bed, breathing and doing fine. In Halmstad, Sweden, about 200 homes are visited and around 780 km are driven each night by caregivers providing nighttime home care [4].

Even though home care services prevent the institutionalization of many individuals [3], there are a number of issues that will likely limit their efficiency and effectiveness in the near future. By the year 2050, about 27% of the European population is expected to be of the age 65 years and above, and in Sweden, older adults will account for 23% of the Swedish population [5]. While many individuals will remain healthy and independent into late adulthood, others will be highly dependent on informal or professional care [6]. Consequently, the demand for home care services will drastically increase, and as it currently stands, the healthcare system is not prepared to address these demands, mostly due to the shortage of professionals specializing in geriatric care [7] and nighttime caregiving [3,8].

When it comes to nighttime home care, not all individuals receiving such services are actually in need of it, because they are still relatively independent and can use the social alarm device to request assistance if it is ever needed [4]. Furthermore, some care beneficiaries report being awakened by the night patrol supervision visit, and these individuals often trigger their alarm within minutes after a supervision visit [4].

Smart home technologies can enhance or complement home health care and have been shown to be integral parts of a cost-effective healthcare system [9,10]. A smart home provides a home-based infrastructure that integrates network-enabled devices with different capabilities to offer advanced functionalities to the residents. Traditionally, smart homes have included solutions to enhance the comfort and safety of residents, as well as systems to manage and conserve energy [11,12]. However, over the past several years, there has been an increased interest in using the pervasive infrastructure of smart homes to support aging in place and AAL.

Systems targeting aging in place and AAL aim to support older or disabled individuals with services that: (1) promote healthier lifestyle and enhanced quality of life; (2) enable early disease detection and treatment compliance; (3) support informal and professional caregiving; and (4) enable individuals to live independently for a longer time in their own homes [13,14].

The current practice of assessing the nature of chronic diseases is limited to clinic-based assessments scheduled at discrete points in time, and the management of illnesses is limited to a few medical visits and to self-reports [15]. The collection and analysis of functional, safety, security and physiological parameters, as well as cognitive and social support are the most common smart home applications in healthcare [16].

In-home health monitoring provides accurate and reliable long-term data to support better decision making, better understanding of aging and illnesses, the prevention and management of chronic diseases, healthier attitudes and behaviors and the conservation of healthcare resources [15,17]. Moreover, the long-term storage of health-related information enables the use of data mining methods that can reveal unknown patterns or relationships that can indicate the onset of a health-related problem [18].

Smart homes and AAL systems are complex to build, use and maintain [19]. One factor contributing to such complexity is the inherent diversity that is characteristic of smart homes and that leads to technical issues related to personalization, integration, interoperation, extensibility and dependability. Individuals have needs, preferences, habits and adverse health conditions that differ and evolve over time. Home environments also differ, and heterogeneous technologies, such as sensors and actuators, are employed in these systems. These distinct devices are provided by different manufacturers, and they operate and communicate with different standards and protocols. Thus, there is no universal arrangement of devices to fit every home environment.

The acceptance of smart homes and AAL systems is also an issue and is directly linked with the system's ability to address an individual's evolving needs, as well as their concerns for privacy, security and dependability [20]. Regarding privacy, not all individuals will accept technologies that monitor all aspects of their lives. Cameras, for example, are perceived as invasive technologies. Moreover, collected data from such systems are very sensitive. In the same way that data analysis of stored data can predict the onset of a health-related problem, data analysis could also predict the predisposition of a person to commit a crime [21]. As a consequence, there are different issues related to data security, such as who is going to use or have access to the data and how and where the data is going to be processed, stored and used. Concerns related to dependability are associated with trust, e.g., can users rely on the system and what if the system stops working altogether.

### 1.2. Related Work

A number of smart homes and AAL projects have been developed over the past several years (reviewed in [22,23]) along with the technical infrastructures that serve as foundations for AAL applications [24]. Although some architectural aspects are common among existing smart environments and AAL platforms, there is still no widely adopted method for developing these systems [25]. Different software architectures have been proposed for the smart environment and AAL domains, including service-oriented architecture (SOA), service-oriented device architecture (SODA), peer-to-peer architecture (P2P), event-driven architecture (EDA), component and connector (C2), multi-agent system (M.A.S) and blackboard. However, as discussed in [26], none of them can perfectly fit the requirements for AAL systems, specifically the requirement for integration [26].

Sensors and actuators provide the means for perceiving and controlling the environment. These devices, among others, are provided by different manufacturers and operate and communicate through different standards and protocols. The open-service gateway initiative (OSGi) framework is commonly used to abstract and integrate devices, such as sensors and actuators, as well as to create service-oriented applications.

Several projects have adopted platforms or middlewares based on SOA and built on top of the OSGi service framework. The Gator Tech Smart House [27], PERSONA (PERceptive Spaces prOmoting iNdependent Aging) [28], SOPRANO (Service Oriented PRogrammable smArt enviroNments for Older Europeans) [29] and universAAL (UNIVERsal open platform and reference Specification for Ambient Assisted Living) [30] are examples of smart homes and AAL projects based on SOA and OSGi.

**Figure 1.**
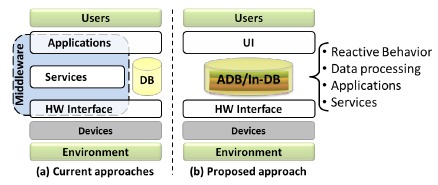
(**a**) Existing infrastructures supporting smart environments and AAL systems perform data processing at different layers; (**b**) in the proposed database-centric architecture, the reactive behavior and data processing are integrated and performed within the database management system (DBMS). Notation: ADB, active database; DB, database; In-DB, in-database processing, HW, hardware; UI, user interface.

Current infrastructures supporting smart environments and AAL solutions typically implement the domain logic along with methods for data analysis, data mining and machine learning, as well as the mechanisms for security and privacy at the application, service or middleware layers (Figure 1a).

Modern DBMSs—such as PostgreSQL [31]—provide mechanisms that can be utilized to address important requirements for data processing and analysis, security, privacy, dependability, extensibility and scalability in smart home and AAL systems. Such mechanisms have not been explored by current smart environments and AAL infrastructures that employ DBMSs exclusively for data storage and retrieval.

### 1.3. Approach and Contribution

In response to the challenges previously described and as an alternative to current approaches, this work presents a database-centric system architecture that exploits mechanisms provided by DBMSs to support the development of AAL applications. The aim is to push the reactive behavior and the data processing, which are commonly implemented at different software layers within existing architectures, into the DBMS (Figure 1b).

This work exploits active databases to detect and respond to events taking place in the home environment, such as bed-exits. The extensibility capabilities of DBMSs, which are mostly provided by user-defined functions, are also explored in this work to perform in-database processing. This means that the domain logic (e.g., for detecting and responding to emergencies) is integrated into the DBMS itself. Three distinct AAL services—bed-exit detection, discovery of common room transitions and behavior modeling—are implemented using the proposed database-centric architecture and are evaluated with a dataset collected in real homes from older individuals living alone.

Active databases and in-database processing avoid transferring sensitive data outside the database. Moreover, the domain logic is centralized into the DBMS and managed on the fly.

The remainder of this paper is organized as follows. An overview of the capabilities of DBMSs, other than data management, is presented in Section 2. Section 3 describes a motivating scenario for AAL applications. The proposed database-centric architecture and its main components are presented in Section 4 and are evaluated with the development of three home-based healthcare services in Section 5. Conclusions are presented in Section 6.

## 2. Capabilities of Database Management Systems

Traditionally, DBMSs are passive components in architectures supporting smart environments and AAL solutions and are employed exclusively to store and manage data for later retrieval. The SQL (Structured Query Language) language is used solely for specifying the database schema and for accessing or manipulating data. However, DBMSs can do much more than data management.

DBMSs incorporate active rule processing mechanisms in the form of database triggers. These provide an event-driven architecture that enables the DBMS to monitor and react to events taking place inside or outside of the database, for example, to enforce referential integrity or to react to sensor data being inserted into the database, respectively.

Moreover, DBMSs enable developers to implement new procedures, functions and data types that are stored within the DBMS. DBMSs also promote mechanisms for controlling security and privacy. DBMSs are very dependable systems, mostly due to high-availability, robustness and reliability, and they enable changes in the domain logic, reactive behavior and security policies to be managed on the fly. This facilitates the system's scalability, maintainability and personalization, because changes in software applications connected to the DBMS are not required [32].

Although the aforementioned capabilities are present in the most widely-used commercial (e.g., Oracle, Microsoft SQL Server and IBM DB2) and open-source (e.g., PostgreSQL and MySQL) DBMSs, the database-centric architecture presented in this work focuses only on the capabilities provided by PostgreSQL [31].

### 2.1. Active Databases

The SQL language enables the creation of database triggers that provide an in-database event-driven architecture to detect and respond to events, such as data manipulation operations, such as table insertions and updates. Database triggers are event-condition-action (ECA) structures—commonly referred to as active rules—meaning that when an event occurs, the condition is evaluated, and if it holds, an action is executed. The action can be executed before or after a data manipulation operation, for example, after a table insertion and/or update.

DBMSs exploiting active rules are called active databases [33]. An active database can monitor and respond to specific circumstances of relevance to an application in a timely manner [33]. For example, active rules can react to incoming sensor data to control smart environments [34]. Active databases can also prevent client applications from periodically querying (polling) the database for data changes. Periodic polling mechanisms can be inefficient (too many queries due to a short polling interval) and inaccurate (delayed response due to a long polling interval). To notify client applications about the occurrence of a certain event, such as a data change, active database systems can make use of external or built-in inter-process communication mechanisms. Such an approach requires the client application to be always connected to the DBMS and to subscribe to notifications published by the DBMS.

PostgreSQL, for example, provides a built-in asynchronous publish-subscribe mechanism for inter-process communication using the NOTIFY (publish), LISTEN (subscribe) and UNLISTEN (unsubscribe) commands.

### 2.2. SQL Extensions

DBMSs enable the SQL language to be extended with user-defined types (UDTs), user-defined aggregates (UDAs), user-defined functions (UDFs) and stored procedures (SPs). UDTs, UDAs, UDFs and SPs can subsequently be included in SQL statements and queries. Moreover, the actions invoked by database triggers are commonly implemented as UDFs or SPs. UDFs and SPs enable in-database processing and analytics—*i.e.*, the semantics of applications, statistical models and machine learning techniques—to be integrated and performed within the DBMS. SQL extensions, including database triggers, are implemented in SQL language or using database vendor-specific procedural languages, such as PL/pgSQL (procedural language for PostgreSQL), Python variants [31] and C language.

PostGIS [35], for example, is a free and open source database extension that adds spatial and geographic objects for PostgreSQL. Advanced algorithms, such as methods for statistical analysis and machine learning, can also be integrated into modern DBMSs. For example, MADlib [36] is an open-source library that adds in-database analytical capabilities for PostgreSQL. The MADlib library supports established methods for supervised learning (linear and logistic regression, decision trees and support vector machines), unsupervised learning (k-means clustering and association rules) and descriptive statistics, and it comes with support modules that provide array operators and probability functions among many other methods [36].

Database extensions are stored into the DBMS and are managed on the fly without requiring system restarts. In-database processing facilitates code reuse and maintainability, avoids data movement and improves performance and security. Performing data processing inside the DBMS is more efficient than with external data mining programs [37,38]. The in-database implementation of different statistical models and machine learning techniques, along with their advantages, can be found and are discussed in [37–42].

### 2.3. Security and Privacy

In addition to active databases and in-database processing, which avoid transferring sensitive data from the database to external applications, DBMSs provide other mechanisms to enforce data security and privacy, such as authentication and authorization.

DBMSs support authentication mechanisms that are used to check and confirm the identity of a user, device or software application trying to access database resources. Besides password-based authentication, DBMSs, such as PostgreSQL, enable authentication methods and protocols, such as Lightweight Directory Access Protocol (LDAP) authentication, the Kerberos network authentication protocol, and Secure Sockets Layer (SSL) certificates, among others.

DBMs also support authorization mechanisms that are used to manage and control users' access permissions to database resources. PostgreSQL manages database access permissions using the concept of roles that can be attributed to a DBMS user or to a group of DBMS users [31].

## 3. Motivating Scenario: The “Trygg om natten” (Safe at Night) Project

The “Trygg om natten” (Safe at night in Swedish) project was conducted in Halmstad, Sweden and explored how technology could assist care beneficiaries and caregivers during nighttime supervisions [4]. The study also focused on how technology was perceived by the participants in terms of integrity and acceptance.

The criteria for selecting participants for the project were that individuals had to be beneficiaries of nighttime supervisions, live alone in their own house or apartment without pets and sustain some level of independence, such as for showering, dressing, eating, functional mobility and personal and toilet hygiene. In addition, an approval of the night patrol team was also required. Individuals diagnosed with some type of dementia or not able to give informed consent were excluded.

In total, 15 out of 30 nighttime supervisions beneficiaries (2 men and 13 women) with an average age of 82 years participated in the project. Ten participants lived in apartments and five in houses.

The home of each participant was equipped with five types of sensors (Table 1) that were active from 10 p.m. until 6 a.m. every night for approximately 14 days.

Two data collections were discontinued during the project, one due to the illness of the participant and another because the participant no longer had the need for nighttime supervision.

**Figure 2.**
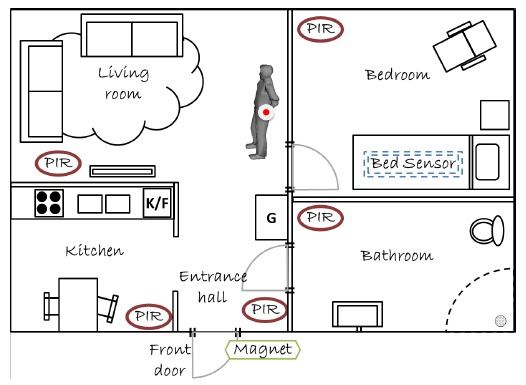
Example of a sensor setup for a given home environment. PIR denotes passive infrared motion sensors; the magnet to capture door openings; the bed sensor to detect bed exits; the resident wears a social alarm. A load cell to measure weight is placed on the top-left leg support of the bed.

Figure 2 illustrates possible placements of different types of sensors within the home environment. The Emfit Bed Sensor was used in the project as the main method to detect bed exits. One strain-gauge load cell was placed at the top-left corner support of the participant's bed to serve as a reference for the Emfit Bed Sensor. Bed entrances and exits, as well as presence in bed, were derived from the measured weight data. Motion sensors in different locations in the home captured human movement in the bedroom, living room, bathroom and kitchen. A magnet sensor installed in the front door monitored whether the front door was opened or closed. The intent with the magnet sensor in the front door was to capture nighttime supervision visits. Except for the load cell, all of the other sensors transmitted the measured data wirelessly to a low-power, fanless, miniature host computer located under the bed.

The load cell was connected to an analog-to-digital converter that was connected to a USB port of the host computer. The study was granted ethical approval from the central ethical review board. One of the outcomes of the study was a set of requirements and specifications for AAL services, particularly those related to nighttime caregiving. The dataset collected during the “Trygg om natten” project was used in this work to evaluate the proposed services.

## 4. Database-Centric Architecture to Support Ambient Assisted Living Systems

This section presents how different DBMSs capabilities fit together in the proposed database-centric system architecture to support smart homes and AAL systems.

**Figure 3.**
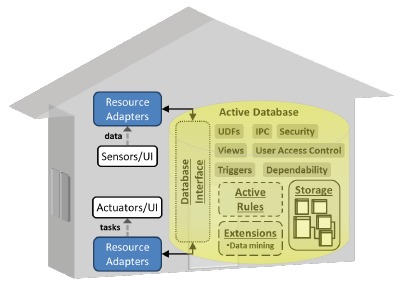
The proposed system architecture, including resource adapters and the active database. Notation: UI, user interface; UDFs, user-defined functions; IPC, inter-process communication.

Figure 3 summarizes the framework in which the proposed system operates and its main components, described in the next subsections.

### 4.1. Resource Adapters

As there is still no adopted standard for communicating with and integrating devices and applications inside smart homes [43], resource adapters have been designed to abstract heterogeneous hardware technologies (sensors and actuators) and software technologies (user interfaces and files) in order to facilitate their integration and interoperation into the system. Resource adapters resemble context widgets and context services [44], but with fewer responsibilities (no data aggregation or peer-to-peer communication). Resource adapters encapsulate the underlying implementation of different communication protocols and abstract resource-specific data formats. Recovery from faults, such as communication disconnections, can also be provided. Resource adapters serve as a gateway between the environment and the DBMS and are implementable in different programming languages, such as C# and Python. Resource adapters stream data acquired by sensors or entered by the user into the database. They also control actuators and user interfaces in response to commands received from the database. Resource adapters communicate with the database through the database interface (Figure 3), and the DBMS employs inter-process communication mechanisms to communicate with resource adapters. Therefore, resource adapters keep an open connection with the DBMS and subscribe to specific event channels.

### 4.2. Active Database

The active database (Figure 3) includes several modules that are used as follows.

#### 4.2.1. Storage

The storage module includes the tables for storing sensor data, processed information and meta-data (location, capabilities and configuration) of the hardware and software resources that are present in the environment. Developers implementing resource adapters do not have access to the internal storage model. They are provided instead with a database interface.

#### 4.2.2. Database Interface

The internal database model is protected from direct access by the database interface module that exposes data access (selections) and manipulation (insertions, updates and deletions) using views and UDFs. Listing 1 shows an example of such an approach.


**Listing 1**. UDF written in PL/pgSQL for inserting converted weight samples into table *weight*.
1.CREATE FUNCTION weight_insert ( adc_out integer , ts timestamp )2.RETURNS boolean AS $3.DECLARE4.  voltage_weight_ratio numeric ≔ −41943.0;5.  weight_sample numeric ;6.BEGIN7.  weight_sample ≔ adc_out / voltage_weight_ratio ;8.  INSERT INTO weight VALUES ( weight_sample , ts );9.  RETURN true ;10.END;11.$ LANGUAGE PLPGSQL


The UDF named *weight_insert* abstracts the insertion into table *weight* and is also used to process the input parameters. The UDF *weight_insert* receives two parameters, the output of the analog-to-digital converter (*adc_out*) and timestamp (*ts*). In Listing 1, Line 7, the voltage-to-weight ratio (*voltage_weight_ratio*) variable is used to convert the readout value (*adc_out*) to weight (*weight_sample*), which is later inserted into table *weight*. Such an approach facilitates changes in the logic, such as in the voltage to weight conversion, because it is performed on the fly and does not require modifications or recompilations of resource adapters.

To notify resource adapters about data changes or events, the active database makes use of built-in mechanisms in PostgreSQL for inter-process communication (NOTIFY and LISTEN commands), and this prevents resource adapters from periodically querying (polling) the database.

#### 4.2.3. Active Rules

The reactive behavior in the system is supported by the active rules module. In conjunction with sensors and actuators, active rules implemented as database triggers (Listing 2) can monitor and react to events happening in the environment.


**Listing 2**. A database trigger monitors when a sequential sample identifier (*sample_id*) of a first in, first out (FIFO)-type of table (*weight_fifo*) wraps around to execute an action (*check_presence_absence*).
1.CREATE TRIGGER weight_fifo_after_insert2.  AFTER INSERT3.  ON weight_fifo4.  FOR EACH ROW5.  WHEN ( NEW. sample_id == 40 )6.  EXECUTE PROCEDURE check_presence_absence ( );


In Listing 2, the ECA rule represented by the trigger *weight_fifo_after_insert* is associated with the table *weight_fifo* and fires after table insertion events. If the condition specified by the Boolean expression in Listing 2 Line 5 is satisfied, the action—*check_presence_absence*—is executed. Because the analog-to-digital samples the load cell at 80 Hz, the trigger fires every half second or every 40th insertion and is intended to detect bed entrances and exits.

#### 4.2.4. Database Extensions

Active rules invoke actions that can be functions added by database extensions, such as MADlib [36], or can be user defined. These functions implement both short-term and long-term types of services. Short-term services are those that respond to simple events, such as generating an alarm indicating a bed exit. Long-term services are defined as services requiring datasets collected over a longer period of time and the analysis of patterns in such data, for example, to gain knowledge about preferences or to detect abnormal behaviors [45]. Because sensitive data are involved in the data processing, implementing the methods for such analysis into the DBMS itself avoids data movement and leads to better performance and security.

#### 4.2.5. Security

Table 2 presents possible access privileges according to different roles in the system (similarly to [46]). The owner can grant or revoke the access privileges of other system users. Software developers creating resource adapters are granted execute permission on specific UDFs within the database interface.

## 5. Experimental Results: In-Database Services Supporting AAL Systems

Three distinct AAL services for home-based health monitoring, inspired by the “Trygg om natten” project (Section 3), are presented and implemented following the proposed architecture.

To accommodate the proposed architecture, a database server was configured in a computer running CentOS 6.4 with PostgreSQL (version 9.2.3) and the MADlib [36] library extension. To implement the proposed services, additional tables were created to store temporary and derived data, such as descriptive statistics and transition matrices. A separate computer running MS Windows 7 hosted resource adapters (implemented in C#) that read the measurements from the “Trygg om natten” dataset files to the corresponding database. The dataset from a single care beneficiary, who was an active man, living alone in his own apartment and receiving daytime home care services and nighttime supervision, was selected to present the implementation results.

### 5.1. Detection of Bed Presence and Absence

**Figure 4.**
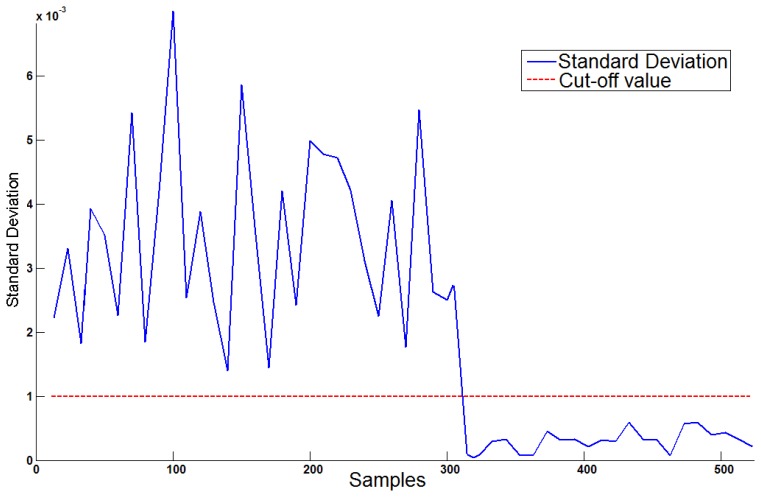
A cut-off value can separate the standard deviation of the measured weight signal into in-bed signals and out-of-bed signals.

A service to detect presence in bed can enable the night patrol team to remotely check if individuals are in bed, so as not to disturb their sleep. Voluntary and involuntary body movements create disturbances in the load cell signal that are not present when the bed is unoccupied or is loaded with static weight. Figure 4 presents the standard deviation of a weight signal measured by a load-cell sensor in the moments before the person left the bed. By analyzing the measured weight and its standard deviation, a method to detect the presence or absence of a person in bed can be implemented as an active rule (trigger) that monitors the table in which the measured weight is stored.

The active rule triggers every half second and invokes a UDF that checks for bed exits and entrances. The condition (Equation (1)) for the detection consists of checking intervals in which the median (*m_w_*) and the standard deviation (*σ_w_*) of the weight signal are greater than the respective estimated thresholds for the mean value (*O_m_w__*) and standard deviation (*O_σ_w__*).

(1) $$\text{Presence}=((\sigma_{w}\geq O_{\sigma_{w}})\text{AND}(m_{w}\geq O_{m_{w}}))$$

The mean value and standard deviation of the weight signal are calculated with a moving window with the last 40 inserted weight samples (approximately half a second or half of the signal sampling rate, which was 80 Hz). Smaller window sizes can lead to high granularity that makes it difficult to find the separating threshold, and larger window sizes can delay the detection of bed entrances and exits.

A method for finding a threshold in a signal (*i.e.*, binarizing) is the Otsu algorithm [47], which maximizes the between-cluster distance when dividing the distribution of values into two clusters, for example, the presence and absence clusters. For each individual, corresponding thresholds have been calculated.

For the selected individual, 27 bed presences and 16 bed absences were detected by the active rule based on measured weight. To identify true and false positives, the dataset containing load cell signals was manually labeled and served as a baseline for comparison.

All bed presence and absence detections were validated as true positives. Bed absence detections outnumbered bed presence detections, because on many occasions, the individual left the bed after the sensors became inactive at 6 a.m.

**Figure 5.**
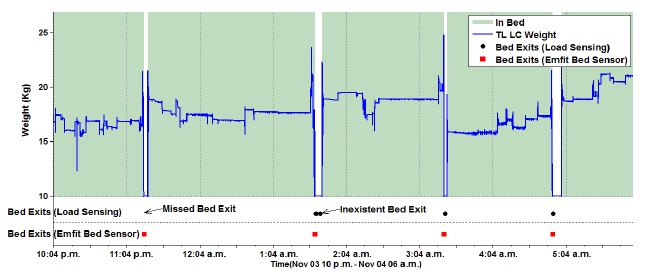
Bed entrances and exits are accurately detected by the active rule, while the bed-exit detection provided by the Emfit Bed Sensor misses bed exits or generates nonexistent bed exits. Because the sensors were active from 10 p.m. until 6 a.m., it was not possible to detect when the individual went to bed or when he left. TL LC denotes top-left load cell.

The proposed approach to detect bed exits and entrances also detected more bed-exit events than the bed-exit detection provided by the Emfit Bed Sensor. Figure 5 presents one missed and one nonexistent bed exit using the Emfit Bed Sensor. For the same individual, the Emfit Bed Sensor missed approximately 60% of all bed exits. Such a mismatch might be caused by the antidecubitus mattress that the individual was using to prevent and treat pressure sores.

The overall approach avoids raw load cell data, which exposes private and sensitive information, from being transferred and processed outside of the DBMS. In this system, several resource adapters can subscribe to the service and are notified when bed entrances and exits are detected.

### 5.2. Common Event Transitions during the Night

The purpose of this service is to enable the detection of anomalies by discovering simple associations between presence detections in the bathroom, living room, kitchen, entrance hall and bed. Strong associations indicate common room transitions and room activity, and deviations from such associations can enable the detection of anomalies.

A method for finding such expected patterns in sequences of events (*i.e.*, sequential data mining [48]) is by estimating the probability *p*(*e_y_*∣*e_x_*) of one event *e_x_* being followed by another type of event *e_y_* (similar to [49]). By considering only the previous detected event, a transition matrix can be computed online for each individual using an active rule. Each element in the transition matrix *P* contains the probability of event *e_i_* being followed by event *e_j_*, and this is denoted as *P_ij_*(*e_j_*∣*e_i_*), which is also referred to as the confidence in association rules [50]. The transition matrix can be visualized as a graph by plotting associations over a certain confidence threshold.

An active rule monitors incoming events from all sensors (Table 1 in Section 3) and updates the transition matrix table, which describes the transition probability of events happening during the night. The computation of statistics, such as the mean and standard deviation of the transition time between two events, is also triggered by the rule. Bed-exit events generated by the previously proposed active rule were used due to the higher accuracy than bed-exit events detected by the Emfit Bed Sensor (Figure 5 in Section 5.1).

**Figure 6.**
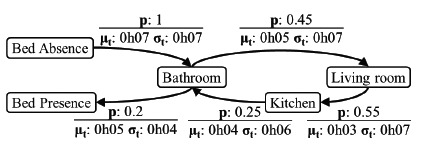
Transition probabilities (*p*) of events for a confidence threshold of 0.2. Mean (*μ_t_*) and standard deviation (*σ_t_*) of the transition time (normally distributed).

Figure 6 presents likely transitions of events in the home environment of the selected subject. An observation from the figure is that when the observed individual leaves the bed, the most likely event is a visit to the bathroom. Such a transition takes an average of 7 min with a standard deviation of 7 min.

The knowledge provided by the transition matrix can be used to detect anomalies during future nights. Anomaly detection mechanisms can also be implemented with active rules. Because health-related conditions evolve over time and because health changes might not be evident in the short-term, the amount of stored data to be processed increases by a large amount every day. Therefore, in-database sequential data mining avoids transferring stored long-term data to external data analysis tools to update transition probabilities.

### 5.3. Modeling of Early Night Behavior Using Decision Trees

Another way to model transitions is with a service that models typical sensor triggering transitions over a certain time span during the night. Such a service could help to discover changing trends in the level of independence of the individual being monitored.

For this service, a decision tree using the C4.5 implementation in MADlib was trained with data from a single individual to discriminate between the time period from 10 p.m. to midnight (TPI denotes Time Period I) and the period from midnight to 6 a.m. (!TPI denotes not Time Period I). The training data consisted of 15 features that were computed for each observation by processing a sliding window with a width of 20 min over the 14 days of collected data. No feature selection has been applied due to the rather low number of features used. This process resulted in training data with approximately 300 observations.

The events in the collected data are denoted as bathroom (Ba), kitchen (K), hallway (H), and living room (L), and each event represents activity in a certain room. Other events include inactivity registered from the wearable inertial sensor (I), door openings (D) and bed entrances and exits (Bin and Bout, respectively), which are computed using the proposed active rule for detecting bed entrances and exits. The features used in the calculations are the type of sensors that fired in the last four events and are denoted as *event at time t*. The transition time between the four last events for the window is computed as *Et*(*t*, *t* − *k*), where *k* is the number of previous events. The number of each type of event and the lack of events (denoted by N) in a window are also computed.

**Figure 7.**
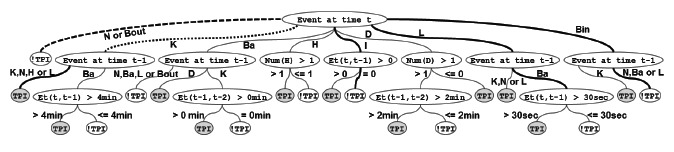
A decision tree distinguishes different time periods during the night. Notation: Ba, bathroom; K, kitchen; H, hallway; L, living room; I, inactivity; D, door openings; Bin, bed entrances; Bout, bed exit; N, lack of events. TPI, Time Period I; !TPI, not Time Period I.

The generated decision tree for the same individual is shown in Figure 7. Thick edges represent where the majority of data points were concentrated. The tree hierarchy reflects variable importance. Figure 7 illustrates (by hierarchy and bold edges) that the last occurring event is important. An example of this is the lack of events (N) in a window, and the presence or absence in bed were the most informative, while discriminating between TPI and !TPI.

One interpretation of the model is illustrated by the dashed edge from the root node. This link revealed that the individual was more likely to be active during the modeled time period TPI than the rest of the night (!TPI). Moreover, the dotted edge shows that the individual was active in the kitchen, hallway and living room during TPI. In order to validate the decision tree model, a 10-fold cross-validation was performed, and a mean accuracy of 81% was achieved. The accuracy shows that, despite the complexity of human behavior, the model is able to explain key features of the early night that could be used when analyzing deviations in long-term trends.

Similar to updating transition probabilities, in-database retraining of decision trees avoids data movement, and this promotes privacy. The processing time to create or update a probability matrix and to train a decision tree can be negligible for a small dataset, but quite significant for a dataset containing months of stored data.

## 6. Conclusions

This work has shown how different capabilities of DBMSs (e.g., triggers, user-defined functions and existing database extensions for in-database analytics) fit together in a database-centric architecture intended to support the development of home-based healthcare applications. The proposed software architecture represents an alternative to existing platforms supporting the development of smart homes and AAL systems.

DBMSs are mature and dependable technologies and provide mechanisms that can address the processing, security, privacy and personalization requirements of smart homes and AAL systems. These mechanisms, however, are not fully exploited in current smart home and AAL infrastructures.

In the system design presented here, database triggers are used to detect and respond to events taking place in the home environment. The event-driven architecture provided by active databases makes it possible to implement an in-database service to monitor an individual's presence or absence in bed, as well as to discover common room transitions and deviations during the night.

User-defined functions are exploited to perform in-database processing, *i.e.*, the domain logic is integrated into the DBMS itself. A database interface created with user-defined functions and views protects the internal database model against direct access. Existing DBMS extensions for data mining, such as MADlib, enable the development of services to model early night behaviors. Database roles can promote security by controlling user access to database resources, such as tables that contain private data.

The proposed and implemented AAL services, which have been validated with a dataset collected in real homes, reside within the database and avoid exporting sensitive data to external data analysis tools.

Therefore, active in-database processing avoids data movement from the DBMS to external applications. Such an approach can lead to improved performance, security and privacy while still benefiting from the on the fly management capabilities of DBMSs. Centralizing the domain logic into the DBMSs reduces code duplication, promotes code reuse and facilitates system maintenance and adaptability as the environment and individual needs evolve.

Although these are important system properties supported by the presented database-centric platform, the proposed approach requires developers to have knowledge of relational DBMSs, their features, such as for security, and procedural programming languages, such as PL/pgSQL and PL/Python [31], that extend the SQL standard.

Even though resource adapters keep an open connection with the DBMS to subscribe to notifications from the database, this is not a limitation, because resource adapters, for example running on battery powered devices, can connect and disconnect to the DBMS when necessary.

Up to now, besides the results reported in this work and in [1], the presented database-centric architecture has been used to develop a smart bedroom [34] and to integrate an autonomous mobile robot to such a smart environment [51]. Future work encompasses developing, deploying and evaluating smart environments that encompass a whole home environment and that provide actuation services to improve comfort, independence and medical care using the presented database-centric platform. To facilitate interoperation, semantic description for the environment, devices and user activities will also be investigated.

## Figures and Tables

**Table 1.** Sensors used in the “Trygg om natten” (Safe at night) project [4].

**Type**	**Purpose**	**Quantity**	**Output**
Passive infrared (PIR)	Capture human motion	3–5	Binary
Quasi-electric film (Emfit)	Capture bed exits	1	Binary
Magnetic	Capture door openings	1	Binary
Inertial sensor	Capture human activity (wearable)	1	Binary
Load cell	Reference for the Emfit sensor	1	24-bit value

**Table 2.** Access privileges according to different roles.

**Role**	**Access Level**			

	**View**	**Add**	**Modify**	**Administer**
**Owner**	X	X	X	X
**Family**	X	X	X	X
**Healthcare**	X	X	X	
**Other users**				
**Devices**		X		
